# Patterns of Nutrient Intake in Relation to Sarcopenia and Its Components

**DOI:** 10.3389/fnut.2021.645072

**Published:** 2021-04-27

**Authors:** Amir Bagheri, Rezvan Hashemi, Ramin Heshmat, Ahmadreza Dorosty Motlagh, Ahmad Esmaillzadeh

**Affiliations:** ^1^Department of Community Nutrition, School of Nutritional Sciences and Dietetics, Tehran University of Medical Sciences, Tehran, Iran; ^2^Department of Geriatric Medicine, Ziaeian Hospital, Tehran University of Medical Sciences, Tehran, Iran; ^3^Chronic Diseases Research Center (CDRC), Endocrinology and Metabolism Population Sciences Institute, Tehran University of Medical Sciences, Tehran, Iran; ^4^Obesity and Eating Habits Research Center, Endocrinology and Metabolism Molecular-Cellular Sciences Institute, Tehran University of Medical Sciences, Tehran, Iran; ^5^Department of Community Nutrition, Isfahan University of Medical Sciences, Isfahan, Iran

**Keywords:** nutrient patterns, sarcopenia, muscle mass, muscle strength, gait speed

## Abstract

**Background:** Despite the associations between individual nutrients and sarcopenia, we are aware of no information about the link between patterns of nutrient intake and odds of sarcopenia and its components. The present study aimed to examine the association between nutrient-based dietary patterns and sarcopenia and its components among the Iranian adult population.

**Methods:** In this population-based, cross-sectional study, we enrolled 300 elderly adults (150 men and 150 women) aged ≥55 years by using a cluster random sampling method. Dietary intakes of the study population were assessed using a validated food frequency questionnaire. Principal component analysis was conducted to derive nutrient patterns based on a daily intake of 33 nutrients. Muscle mass, muscle strength, and gait speed were measured according to standard methods. Sarcopenia and its components were defined based on the European Working Group on Sarcopenia.

**Results:** Three major nutrient-based dietary patterns were identified: (1) the “pro-vit pattern” that was high in pantothenic (B5), cobalamin (B12), calcium, protein, phosphor, riboflavin (B2), zinc, cholesterol, saturated fat, folate, niacin (B3), selenium, vitamin D, vitamin K, and vitamin A; (2) the “anti-inflammatory” pattern, which was rich in polyunsaturated fat, monounsaturated fat, copper, vitamin E, omega-3, magnesium, iron, pyridoxine (B6), sodium, and caffeine; and (3) the “carbo-vit” patternm which is characterized by high intake of fructose, glucose, dietary fiber, biotin, potassium, thiamin (B1), vitamin C, and chromium. After adjusting for confounders, subjects in the top tertile of the anti-inflammatory pattern had lower odds of sarcopenia (OR 0.25; 95% CI 0.10–0.63) and low muscle strength (OR: 0.46; 95% CI: 0.22–0.96) than those in the bottom tertile. Greater adherence to the carbo-vit pattern was inversely associated with the odds of low gait speed (OR: 0.46; 95% CI: 0.235–0.93).

**Conclusion:** Major nutrient-based dietary patterns were significantly associated with sarcopenia and its components. Further studies are required to confirm our findings.

## Introduction

Sarcopenia is a progressive muscle disease that is described as a combination of low muscle quality and physical performance. It is associated with chronic diseases, disability, falls, poor quality of life, and mortality (1, 2). Also, sarcopenia increases the costs of health care systems significantly (1). The prevalence of this disease varies according to the definition. It is prevalent in 16.5–32.5% of the Iranian population (3).

A low-quality diet is one of the major contributing factors to sarcopenia and muscle weakness (4). High intake of proteins, vitamin D, vitamin E, potassium, magnesium, phosphorus, iron, vitamin K, and omega-3 has been shown to preserve muscle mass (4–6). It must be kept in mind that people are not consuming single nutrients (7). Moreover, effects from single nutrients may be too small to be detectable, and assessing the pattern of nutrient intake that considers whole nutrient intake may show a significant association with the risk of chronic diseases (8, 9). Assessment of nutrient intake patterns may provide a better and more general insight into the diet–disease relationship (8). Despite earlier investigations on the relationship between some dietary patterns, including Mediterranean or healthy dietary patterns, and sarcopenia (7, 10), we are aware of no study linking the pattern of nutrient intake and sarcopenia. However, nutrient patterns have been assessed in relation to several other chronic conditions, including psychological disorders (11), obesity (12), metabolic syndrome (13), and some cancers (14).

Finding a nutritional approach to decline muscle wasting and maintain its performance in elderly people is of high priority. Given the growing number of elderly people in developing countries along with the nutritional transition from healthy foods to unhealthy diets and limited evidence about diet–sarcopenia associations, this cross-sectional study was done to identify major nutrient-based dietary patterns in relation to sarcopenia and its components in a group of Iranian people.

## Participants and Methods

### Participants

We conducted this population-based cross-sectional study from May to October 2011 in Tehran, Iran. A detailed report on the sampling method and data-collection procedure has been published previously (15, 16). We used the formula suggested for cross-sectional studies. According to the study of Thomas the standard deviation of appendicular skeletal muscle mass (ASM) in women was 1.9 and in men was 3.4 kg (17). Therefore, the sample size was calculated to be 29 in women and 30 in men in each age group. Finally, 30 men and 30 women in each age category (55–59 y, 60–64 y, 65–69 y, 70–74 y, and over 75 y) were examined. Finally, a total sample of 300 people (150 women, 150 men) enrolled in the study (study power: 80%, design effect: 1.2, α = 5%). We used the cluster random sampling method to recruit individuals in district six of Tehran. The head of each cluster was selected based on a 10-digit postal code. We recruited participants aged ≥ 55 years with the ability to move without crutches, a walker, or assistive devices and those without any active cancers (based on self-reported data). Moreover, we did not include people with artificial limbs or limb prostheses and those with a history of debilitating disease (e.g., congestive heart failure) in our study. The study protocol was approved by the Tehran University of Medical Sciences Ethics' Committee. First, the study goals were clarified to all participants, and then they were requested to complete a written informed consent before data collection.

### Dietary Intake Assessment

A block-format 117-item food frequency questionnaire (FFQ) was used to assess the usual dietary intakes of study participants; the validity and reliability of this questionnaire is reported in previous studies (15, 18). This FFQ included a list of foods with a specific portion size. Participants were able to report their consumption frequency based on a daily, weekly, or monthly basis for each food item. The questionnaire was filled in by a trained nutritionist through a face-to-face interview. Then, we converted the frequency of each food item to grams per day by considering the household measures of portion sizes. Finally, nutritionist IV software with a modified food composition database of the U.S. Department of Agriculture was used to calculate daily energy and nutrient intake of each participant. It should be noted that we did not use the food composition data set of Iran because it is from 40 years ago with an incomplete list of foods and nutrients. Therefore, the USDA food composition table was used; however, for Iranian local foods, we modified the database with the Iranian food composition data.

### Assessment of Sarcopenia

Sarcopenia was defined by considering the combination of both low muscle mass and low muscle function (either strength or performance) according to the European Working Group on Sarcopenia (EWGSOP) definition (19). Muscle mass was measured as the ratio of an individual's total lean mass of legs and arms (also named ASM) to their squared height (ASM/height^2^) (20). We used a DXA scanner (Discovery W S/N 84430) to calculate ASM. Then, low muscle mass was defined as the amount of muscle mass <5.45 (kg/m^2^) for women and 7.26 (kg/m^2^) for men according to EWGSOP (19).

A handgrip test was used to assess muscle strength. The handgrip test was calculated by a pneumatic tool that is a squeeze bulb dynamometer (c7489-02 Rolyan) calibrated in pounds per square inch (psi). We measured the handgrip strength (maximum voluntary contractions) three times for each right and left hand with a 30-s rest in between measurements. The average measurements of both the participant's hands were considered as their muscle strength. Then, handgrip strength <30 kg for men and <20 kg for women was considered to be low muscle strength (21). A four-meter walk gait speed test was applied to calculate muscle performance (19). If participants had gait speeds <0.8 m/s, they were categorized as low muscle performance (19).

### Assessment of Other Variables

A pretested questionnaire was used to collect general characteristics of participants, including age, sex, socioeconomic status, medical history, medication use, smoking habits, and alcohol consumption. A trained interviewer examined the physical activity level by using the short form of the International Physical Activity Questionnaire (IPAQ), which has been validated previously (18). Then, measures of physical activity for each participant were expressed as metabolic equivalent-hour per week (MET-h/week) according to IPAQ's guideline (22). A digital scale was used to measure weight while participants were minimally clothed. Height was measured by a wall tape meter in a standing position without shoes. Waist circumference was measured in the middle of the lower rib margin and iliac crest. Weight (kg) divided by height squared (m^2^) was used to calculate body mass index (BMI).

### Statistical Analysis

Principal component analysis was conducted to determine major nutrient-based dietary patterns based on 33 components. The following nutrients were included in the factor analysis: protein, total dietary fiber, glucose, fructose, total saturated fatty acids (SFAs), total monounsaturated fatty acids (MUFAs), total polyunsaturated fatty acids (PUFAs), cholesterol, vitamin B12, vitamin A, vitamin D, vitamin E, vitamin K, thiamin, riboflavin, niacin, pantothenic acid, pyridoxine, biotin, folate, vitamin C, caffeine, sodium, potassium, phosphorus, magnesium, iron, selenium, calcium, chromium, copper, zinc, and omega-3. The Kaiser Meyer Olkin (KMO) test was used to determine whether the different nutrients were appropriate for principal components or not (KMO test = 0.88). Based on the scree plot, we retained factors with an eigenvalue higher than two as a major nutrient pattern. Varimax rotation was used to obtain independent, nutrient-based dietary patterns. Then, each participant received a factor score for each recognized pattern. To label each major nutrient-based dietary pattern, we considered the factor loading of 0.5 or above; however, we presented all nutrients with a factor loading of 0.2 or above in each nutrient pattern. Finally, we categorized participants into tertiles based on nutrient pattern scores instead of quartiles or quintiles due to avoiding a low number of people in each category. General characteristics of study participants across tertiles of nutrient-based dietary pattern scores were compared using one-way ANOVA and chi-square, where appropriate. The general linear model was used to compare age-, sex-, and energy-adjusted dietary intakes of participants across tertile categories of nutrient-based dietary pattern scores. Prevalence of sarcopenia (yes/no), low muscle mass (yes/no), low muscle strength (yes/no), and low gait speed (yes/no) across tertile categories of nutrient-based dietary patterns were assessed by chi-square. The general linear model was applied to compare means of muscle mass [ASM/h^2^] (kg), muscle strength (psi), and gait speed (m/s) across tertile categories of nutrient-based dietary pattern scores in crude and age-, sex-, and energy-adjusted models. Logistic regression assumptions, including assumptions of normality, homogeneity of variance, homogeneity of variance-covariance matrices, the absence of multicollinearity, and the absence of auto-correlation were met. Then, we conducted multivariable logistic regression to find the association between nutrient-based dietary patterns and sarcopenia (yes/no), low muscle mass (yes/no), low muscle strength (yes/no), and low gait speed (yes/no). In these analyses, we controlled for several confounders. Age (continuous), sex (male/female), and energy intake (kcal/d) were adjusted for in the first model. Then, further adjustments were done for physical activity (MET-h/wk), smoking (yes/no), alcohol consumption (yes/no), medication use (statin, corticosteroid, estrogen, testosterone), and positive history of chronic diseases (yes/no). In all these analyses, the lowest tertile was considered as the reference category, and the odds ratio for sarcopenia in other categories was calculated. We considered the tertile categories as an ordinal variable to calculate the linear trend of odds ratios across tertiles of the nutrient-based dietary pattern scores. All analyses were done using SPSS (version 26). *P*-values were considered significant at <0.05.

## Results

We identified three major nutrient-based dietary patterns using principal component analysis (Table 1). The KMO test was 0.88, showing the adequacy of the sample size. The pro-vit nutrient pattern was high in pantothenic (B5), cobalamin (B12), calcium, protein, phosphorous, riboflavin (B2), zinc, cholesterol, saturated fat, folate, niacin (B3), selenium, vitamin D, vitamin K, and vitamin A. The anti-inflammatory nutrient pattern was characterized by high intakes of polyunsaturated fat, monounsaturated fat, copper, vitamin E, omega-3, magnesium, iron, pyridoxine (B6), sodium, and caffeine. The carbo-vit nutrient pattern was characterized by high consumption of fructose, glucose, dietary fiber, biotin, potassium, thiamin (B1), vitamin C, and chromium. Overall, these nutrient-based dietary patterns explained 68% of the variance.

**Table 1:** Component of the nutrient-based dietary patterns.

**Nutrient**	**Components**		
	**Pro-Vit**	**Anti-inflammatory**	**Carbo-Vit**
Pantothenic	**0.873**	0.245	0.271
Cobalamin (B12)	**0.850**		
Calcium	**0.848**		0.294
Protein	**0.842**	0.405	0.269
Phosphor	**0.837**	0.420	0.278
Riboflavin (B2)	**0.799**	0.245	0.470
Zinc	**0.766**	0.520	0.229
Cholesterol	**0.681**	0.282	
Saturated fat	**0.611**	0.549	
folate	**0.586**	0.494	0.425
Niacin	**0.580**	0.509	0.403
Selenium	**0.525**	0.281	0.221
Vitamin D	**0.489**		
Vitamin K	**0.426**	0.300	0.266
Vitamin A	**0.412**	0.212	0.302
Poly-fat		**0.861**	
Mono-fat	0.316	**0.839**	
Copper	0.249	**0.751**	0.388
Vitamin E		**0.699**	
Omega-3	0.250	**0.677**	
Magnesium	0.554	**0.638**	0.429
Iron	0.493	**0.636**	0.448
Pyridoxine	0.222	**0.628**	0.525
Sodium		**0.522**	
caffein		**0.495**	
Fructose			**0.925**
Glucose			**0.923**
Dietary fiber	0.245	0.390	**0.845**
Biotin	0.349		**0.825**
Potassium	0.517	0.341	**0.721**
Thiamin	0.464	0.431	**0.665**
Vitamin C	0.275	0.283	**0.546**
Chromium		0.248	**0.276**
Percent of variance explained	**26.8%**	**21.7%**	**19.5%**

*Factor loadings <0.2 were omitted due to simplicity. The bold values show the major nutrient's factor loading in each pattern*.

General characteristics of study participants across tertiles of nutrient-based dietary pattern scores are indicated in Table 2. Subjects with the greatest adherence to the pro-vit nutrient pattern were more likely to be physically active than those with the lowest adherence. Individuals in the highest tertile of the anti-inflammatory nutrient pattern were more likely to be physically active than smokers and those who use alcohol. Greater adherence to the carbo-vit nutrient pattern was associated with higher levels of physical activity. There were no other significant differences across tertiles of nutrient-based dietary pattern scores.

**Table 2:** General characteristics of the study participants across categories of nutrient-based dietary pattern scores.

	**Tertiles of Pro-Vit**			***P*^†^**	**Tertiles of Anti-inflammatory**			***P*^†^**	**Tertiles of Carbo-Vit**			***P*^†^**
	**T_**1**_ (*n* = 100)**	**T_**2**_ (*n* = 100)**	**T_**3**_ (*n* = 100)**		**T_**1**_ (*n* = 100)**	**T_**2**_ (*n* = 100)**	**T_**3**_ (*n* = 100)**		**T_**1**_ (*n* = 100)**	**T_**2**_ (*n* = 100)**	**T_**3**_ (*n* = 100)**	
Age (y)^*^	66.8 ± 7.7	67.3 ± 8.1	66.2 ± 7.3	0.60	67.8 ± 7.9	66.8 ± 7.7	65.7 ± 7.3	0.14	66.7 ± 7.7	66.4 ± 8.1	67.1 ± 7.3	0.80
BMI (kg/m^2^)^*^	27.59 ± 3.7	27.14 ± 4.5	27.4 ± 4.4	0.75	27.32 ± 3.9	27.45 ± 4.3	27.37 ± 4.3	0.97	27.62 ± 4.5	27.12 ± 3.6	27.4 ± 4.4	0.01
PA (MET-h/w)^*^	1129.7 ± 989.1	1104.3 ± 1298.3	1649.5 ± 1821.2	0.01	1063.4 ± 1201.1	1190.3 ± 1478.6	1629.8 ± 1537.7	0.01	1082.6 ± 940.5	1160.5 ± 1565.2	1640.4 ± 1628.9	0.70
Female (%)	35.9	34	30.1	0.43	37.3	35.3	27.4	0.08	34	34.6	31.4	0.75
Alcohol use (%)	37.5	22.5	40	0.28	15	37.5	47.5	0.02	35	37.5	27.5	0.68
Smoking (%)	47.4	21.1	31.5	0.10	13.2	39.4	47.4	0.01	50	26.3	23.7	0.06
Medical history												
Yes (%)	36.5	30.8	32.7	0.85	40.4	28.8	30.8	0.48	28.8	34.6	36.6	0.73
No (%)	32.7	33.8	33.5		31.9	34.3	33.8		34.3	33.1	32.6	
Drug history use												
Sexual hormone (%)	33.3	33.3	33.4	1.00	44.4	22.2	33.4	0.70	22.2	44.4	33.4	0.70
Statin (%)	33.6	38.2	28.2	0.27	30.9	37.3	31.8	0.53	25.5	36.4	38.1	0.80
Corticosteroid (%)	37.5	25	37.5	0.88	37.5	25	37.5	0.88	25	50	25	0.60

* *All values are mean ± SD, unless indicated;*

† *ANOVA for continuous variables and chi-squared test for categorical variables*.

Table 3 shows age-, gender-, and energy-adjusted dietary intakes of study participants across categories of nutrient-based dietary pattern scores. Subjects in the top tertile of the pro-vit pattern had a higher intake of vegetables, dairy products, whole grains, red and white meats, energy, protein, calcium, and folate and a lower intake of carbohydrate and vitamin E than those in the bottom tertile. Greater adherence to the anti-inflammatory nutrient pattern was associated with a higher intake of legumes and nuts, vegetable oils, red meats, energy, fat, vitamin E, and folate and a lower intake of carbohydrates, dairy products, and calcium. Individuals in the highest tertile of the carbo-vit pattern had higher intakes of fruits, vegetables, energy, carbohydrates, dietary fiber, vitamin B6, and folate and a lower intake of sweets and desserts, red meats, fat, and vitamin E than those in the lowest tertile.

**Table 3:** Dietary intakes of study participants across categories of nutrient-based dietary pattern scores^*^.

	**Tertiles of Pro-Vit**			***P*^†^**	**Tertiles of Anti-inflammatory**			***P*^†^**	**Tertiles of Carbo-Vit**			***P*^†^**
	**T_**1**_ (*n* = 100)**	**T_**2**_ (*n* = 100)**	**T_**3**_ (*n* = 100)**		**T_**1**_ (*n* = 100)**	**T_**2**_ (*n* = 100)**	**T_**3**_ (*n* = 100)**		**T_**1**_ (*n* = 100)**	**T_**2**_ (*n* = 100)**	**T_**3**_ (*n* = 100)**	
**Food groups (g/day)**												
Fruits	605.8 ± 27.5	681.7 ± 26.4	665.5 ± 27.9	0.12	609.2 ± 27.2	668.9 ± 26.6	675.5 ± 28	0.17	487.4 ±22.7	598.2 ± 22	866.2 ± 23	<0.01
Vegetables	470.4 ± 25.1	527.1 ± 24.2	565.1 ± 25.6	0.04	462.9 ± 24.8	529.8 ± 24.2	569.7 ± 25.6	0.14	393.1 ±22	477.7 ±21.5	691.6 ±22.4	0.01
legumes and nuts	46.9 ± 3.5	52.8 ± 3.3	56.9 ± 3.5	0.15	46.7 ± 3.4	48.4 ± 3.3	61.6 ± 3.5	0.01	50.4 ± 3.4	56.3 ± 3.3	49.9 ± 3.5	0.34
Dairy products	360.1 ± 25.6	498.7 ± 24.7	819.1 ± 26.2	<0.01	681.9 ± 30.1	524.1 ± 29.3	471.8 ± 30.9	<0.01	547.9 ±31.2	565.9 ±30.4	563.9 ±31.7	0.90
Whole grains	46.1 ± 7.1	55.7 ± 6.8	84.1 ± 7.2	0.01	70.7 ± 7.1	57.4 ± 6.9	57.7 ± 7.3	0.32	51.6 ± 7.1	64.6 ± 6.9	69.7 ± 7.2	0.20
Refined grains	246.9 ± 18.2	268.1 ± 17.5	246.6 ± 18.5	0.61	245.4 ± 18	274.5 ± 17.5	241.6 ± 18.5	0.35	245.7 ± 18	265.1 ± 17.5	250.8 ± 18.3	0.72
Sweets and deserts	6.7 ± 0.9	7.9 ± 0.9	4.6 ± 0.9	0.04	5.1 ± 0.96	6.1 ± 0.94	8.1 ± 0.99	0.13	7.9 ± 0.9	7.4 ± 0.9	4.2 ± 0.9	0.03
Red meats	29.9 ± 3	34.1 ± 2.9	42.2 ± 3.1	0.02	27.7 ± 2.9	36.7 ± 2.8	41.8 ± 3.1	0.05	42.9 ± 2.9	35.6 ± 2.8	27.7 ± 3	0.01
White meats	31.6 ± 3.1	39.2 ± 2.9	45.5 ± 3.1	0.01	37.9 ± 3.1	36.6 ± 3	41.9 ± 3.2	0.47	43.2 ± 3.1	37.7 ± 3	35.4 ± 3.1	0.21
Vegetable oils	11.5 ± 0.9	10.9 ± 0.8	9.5 ± 0.9	0.29	8.9 ± 0.9	10.5 ± 0.8	12.4 ± 0.9	0.03	10.9 ± 0.8	11 ± 0.8	9.9 ± 0.9	0.68
**Nutrients**												
Energy	1857.2 ± 84	2165.1 ±84	2766.3 ± 84	<0.01	1929.6 ± 85.5	2103.8 ±85	2755.2 ± 85.8	0.01	1901.8 ± 85.3	2165.9 ± 85.4	2721.1 ± 85.4	<0.01
Carbohydrate (g/d)	380.6 ± 5.5	367.4 ± 5.3	350.1 ± 5.6	0.01	386.3 ± 5.2	374.8 ±5.1	336.8 ± 5.4	<0.01	349.2 ± 5.4	362.5 ±5.3	386.3 ±5.5	<0.01
Protein (g/d)	72.3 ± 1.4	83.5 ± 1.4	102.3 ± 1.5	<0.01	87.7 ± 1.8	84.6 ± 1.8	85.7 ± 1.9	0.47	86.2 ± 1.8	87.1 ± 1.8	84.8 ± 1.8	0.70
Fats (g/d)	60.8 ± 1.9	60.1 ± 1.8	56.9 ± 1.9	0.37	50.4 ± 1.7	56.1 ± 1.7	71.1 ± 1.8	<0.01	65.6 ± 1.8	59.7 ± 1.8	52.5 ± 1.9	<0.01
Dietary Fiber (g/d)	31.1 ± 0.8	30.5 ± 0.7	28.3 ± 0.9	0.08	29.7 ± 0.8	30.7 ± 0.8	29.5 ± 0.9	0.61	25.1 ± 0.7	28.8 ± 0.7	36.1 ± 0.7	<0.01
Calcium (mg/d)	1039.1 ± 35.9	1250.9 ± 34.6	1726.9 ± 36.6	<0.01	1521.5 ± 42.8	1299.7 ± 41.7	1195.7 ± 44.1	<0.01	1273.1 ± 44.4	1350.5 ± 43.3	1393.3 ± 45.2	0.17
Vitamin E (mg/d)	10.5 ± 0.5	9.8 ± 0.4	7.8 ± 0.5	0.01	6.9 ± 0.4	8.3 ± 0.4	12.9 ± 0.4	<0.01	10.6 ± 0.5	9.2 ± 0.4	8.5 ± 0.5	0.02
Vitamin B6 (mg/d)	2.8 ± 0.1	2.6 ± 0.1	2.4 ± 0.1	0.10	2.7 ± 0.13	2.6 ± 0.12	2.5 ± 0.13	0.58	2.3 ± 0.12	2.5 ± 0.12	2.9 ± 0.13	0.01
Folate (mcg/d)	496.3 ± 11.3	541.3 ± 10.9	593.1 ± 11.6	<0.01	512.3 ± 11.6	541.5 ± 11.3	576.8 ± 12	0.01	479.8 ±10.6	538.3 ±10.3	612.5 ±10.8	<0.01

* *All values are mean ± SE; energy intake is adjusted for age and sex; all other values are adjusted for age, sex, and energy intake*.

† *ANCOVA for all variables*.

The prevalence of sarcopenia and its components across tertile categories of nutrient-based dietary pattern scores are depicted in Table 4. There were no significant differences in the prevalence of sarcopenia and its components across tertiles of the pro-vit and carbo-vit patterns; however, subjects with the greatest adherence to the anti-inflammatory nutrient pattern were less likely to have sarcopenia (*P* = 0.02) and low handgrip strength (*P* = 0.05) than those with the lowest adherence.

**Table 4:** Prevalence of sarcopenia and its components across tertile categories of nutrient-based dietary pattern patterns.

	**Tertiles of Pro-Vit**			P^*^	**Tertiles of Anti-inflammatory**			P^*^	**Tertiles of Carbo-Vit**			P^*^
	**T_**1**_ (*n* = 100)**	**T_**2**_ (*n* = 100)**	**T_**3**_ (*n* = 100)**		**T_**1**_ (*n* = 100)**	**T_**2**_ (*n* = 100)**	**T_**3**_ (*n* = 100)**		**T_**1**_ (*n* = 100)**	**T_**2**_ (*n* = 100)**	**T_**3**_ (*n* = 100)**	
**Prevalence of components of sarcopenia**												
low muscle mass (%)^†^	34.2	33.3	32.5	0.96	35	35	30	0.60	35	32.5	32.5	0.88
low handgrip strength (%)^‡^	31.3	34.4	34.3	0.87	42.7	30.2	27.1	0.05	35.4	26	38.6	0.16
low gait speed (m/s) (%)^§^	34.4	36.9	28.7	0.34	35.2	38.5	26.3	0.08	39.3	33.6	27.1	0.09
Sarcopenia (%)	31.5	42.6	25.9	0.24	46.3	35.2	18.5	0.02	38.9	31.5	29.6	0.62
Means of components												
Muscle mass [ASM/h^2^] (kg)												
Crude	6.5 ± 0.1	6.5 ± 0.1	6.7 ± 0.1	0.43	6.4 ± 0.1	6.6 ± 0.1	6.7 ± 0.1	0.07	6.5 ± 0.1	6.5 ± 0.1	6.7 ± 0.1	0.26
Model 1	6.5 ± 0.08	6.6 ±0.08	6.6 ± 0.08	0.81	6.5 ± 0.08	6.6 ± 0.08	6.6 ± 0.08	0.49	6.6 ± 0.08	6.5 ± 0.08	6.7 ± 0.08	0.25
Hand grip strength (psi)												
Crude	10.8 ± 0.3	10.7 ±0.3	11.5 ± 0.3	0.29	10.2 ± 0.3	10.9 ± 0.3	11.9 ± 0.3	<0.01	11.1 ± 0.3	11.2 ± 0.3	10.9 ± 0.3	0.84
Model 1	11.1 ± 0.2	10.9 ±0.2	11.2 ± 0.2	0.67	10.6 ± 0.2	11.1 ± 0.2	11.4 ± 0.2	0.07	11.1 ±0.2	11.2 ±0.2	10.8 ± 0.2	0.42
Gait speed (m/s)												
Crude	0.8 ± 0.02	0.8 ±0.02	0.8 ± 0.02	0.08	0.83 ± 0.02	0.83 ±0.02	0.86 ±0.02	0.45	0.82 ± 0.02	0.85 ± 0.2	0.85 ± 0.01	0.43
Model 1	0.83 ± 0.02	0.82 ± 0.02	0.87 ± 0.02	0.20	0.85 ± 0.02	0.83 ± 0.02	0.84 ± 0.02	0.85	0.82 ± 0.02	0.85 ± 0.02	0.85 ± 0.02	0.46

* *Obtained from ACNOVA for quantitative variables and chi-square for qualitative variables (P < 0.05 significant)*.

† *Muscle mass lower than 5.45 (kg/m^2^) for women and 7.26 (kg/m^2^) for men were considered low (19)*.

‡ *Muscle strength lower than 20 kg for women and 30 kg for men was considered low (21)*.

§ *Gait speeds lower than 0.8 m/s were considered low (19)*.

*Model 1: Adjusted for energy, age and sex*.

Multivariable-adjusted logistic regression analysis indicating the link between nutrient-based dietary patterns and odds of sarcopenia and its components is provided in Table 5. We did not find any association between adherence to the pro-vit nutrient pattern and odds of sarcopenia either before (OR: 0.79; 95% CI: 0.36–1.71) or after controlling for potential confounders (OR: 0.74; 95% CI: 0.31–1.76). Also, no significant associations were seen between this nutrient pattern and components of sarcopenia. Subjects in the top tertile of the anti-inflammatory nutrient pattern were 67% less likely to have sarcopenia (OR: 0.33; 95% CI: 0.15–0.74) than those in the bottom tertile. The association strengthened in the fully adjusted model (OR 0.25; 95% CI 0.10–0.63). Also, participants in the top tertile of this pattern were less likely to have low handgrip strength in the crude (OR: 0.50; 95% CI: 0.27–0.92) and fully adjusted models (OR: 0.46; 95% CI: 0.22–0.96). Adherence to the carbo-vit nutrient pattern was significantly associated with 47% decreased odds of low gait speed in the crude model (OR: 0.53; 95% CI: 0.30–0.94). This protective association remained significant even after controlling for all potential confounders (OR: 0.46; 95% CI: 0.235–0.93).

**Table 5:** Multivariable-adjusted odds ratios (95% CIs) for sarcopenia and its components across tertile categories of nutrient-based dietary patterns.

	**Tertiles of Pro-Vit**			***P*_**trend**_**	**Tertiles of Anti-inflammatory**			***P*_**trend**_**	**Tertiles of Carbo-Vit**			***P*_**trend**_**
	**T_**1**_**	**T_**2**_**	**T_**3**_**		**T_**1**_**	**T_**2**_**	**T_**3**_**		**T_**1**_**	**T_**2**_**	**T_**3**_**	
Sarcopenia												
*N*	100	100	100		100	100	100		100	100	100	
Crude	1	1.46 (0.72–2.93)	0.79 (0.36–1.71)	0.58	1	0.70 (0.36–1.38)	0.33 (0.15–0.74)	<0.01	1	0.77 (0.38–1.56)	0.72 (0.35–1.47)	0.36
Model 1^†^	1	1.36 (0.66–2.78)	0.69 (0.30–1.60)	0.44	1	0.67 (0.34–1.34)	0.25 (0.10–0.62)	<0.01	1	0.75 (0.36–1.55)	0.62 (0.28–1.37)	0.23
Model 2^‡^	1	1.39 (0.66–2.90)	0.74 (0.31–1.76)	0.55	1	0.65 (0.32–1.326)	0.25 (0.10–0.63)	<0.01	1	0.70 (0.33–1.47)	0.59 (0.25–1.36)	0.22
Low muscle mass^†^												
Crude	1	0.96 (0.54–1.69)	0.92 (0.52–1.62)	0.77	1	1.00 (0.57–1.75)	0.77 (0.44–1.37)	0.38	1	0.88 (0.50–1.55)	0.88 (0.50–1.55)	0.66
Model 1^†^	1	0.89 (0.48–1.64)	0.84 (0.43–1.64)	0.62	1	0.98 (0.54–1.81)	0.64 (0.33–1.25)	0.21	1	0.89 (0.48–1.64)	0.83 (0.43–1.58)	0.56
Model 2^‡^	1	1.03 (0.51–2.07)	1.07 (0.55–2.07)	0.93	1	0.93 (0.49–1.75)	0.54 (0.26–1.10)	0.10	1	0.97 (0.51–1.82)	0.96 (0.48–1.92)	0.92
Low hand grip strength^‡^												
Crude	1	1.15 (0.63–2.08)	1.15 (0.63–2.08)	0.65	1	0.58 (0.33–1.05)	0.50 (0.27–0.92)	0.02	1	0.64 (0.35–1.19)	1.14 (0.64–2.03)	0.65
Model 1^†^	1	1.21 (0.63–2.31)	1.32 (0.65–2.67)	0.43	1	0.51 (0.27–0.96)	0.44 (0.22–0.90)	0.02	1	0.59 (0.31–1.15)	1.29 (0.66–2.52)	0.49
Model 2^‡^	1	0.80 (0.38–1.67)	0.94 (0.47–1.86)	0.55	1	0.50 (0.26–0.98)	0.46 (0.22–0.96)	0.03	1	0.57 (0.28–1.14)	1.27 (0.62–2.58)	0.53
Low gait speed (m/s)^§^												
Crude	1	1.13 (0.64–1.97)	0.74 (0.42–1.32)	0.31	1	1.17 (0.67–2.05)	0.62 (0.35–1.11)	0.11	1	0.75 (0.43–1.32)	0.53 (0.30–0.94)	0.03
Model 1^†^	1	1.15 (0.64–2.07)	0.83 (0.43–1.59)	0.60	1	1.31 (0.73–2.35)	0.78 (0.41–1.50)	0.52	1	0.72 (0.40–1.31)	0.48 (0.25–0.91)	0.03
Model 2^‡^	1	1.17 (0.59–2.32)	1.19 (0.62–2.29)	0.67	1	1.40 (0.76–2.58)	0.99 (0.50–1.97)	0.92	1	0.66 (0.35–1.24)	0.46 (0.235–0.93)	0.03

† *Model 1: Adjusted for age, sex, and energy intake*.

‡ *Model 2: Further adjusted for physical activity, smoking, alcohol consumption, medication use (statin, corticosteroid, estrogen, testosterone), and positive history of disease*.

*^†^Muscle mass lower than 5.45 (kg/m^2^) for women and 7.26 (kg/m^2^) for men were considered low (19)*.

*^‡^Muscle strength lower than 20 kg for women and 30 kg for men was considered low (21)*.

§ *Gait speeds lower than 0.8 m/s were considered low (19)*.

## Discussion

In this cross-sectional study, we found a significant inverse association between adherence to anti-inflammatory nutrient-based dietary patterns and odds of sarcopenia and low muscle strength. Also, the carbo-vit pattern was significantly associated with decreased odds of low gait speed. To the best of our knowledge, this is the first study investigating the association between nutrient patterns and sarcopenia.

Nutrient pattern analysis is a new approach in nutritional epidemiology that is designed to consider all nutrient interactions in a single exposure (23). Although several studies assess the association between major dietary patterns and risk of sarcopenia (24), there is no information available on the link between patterns of nutrient intake and sarcopenia. Sarcopenia and its components are closely related to nutritional status (25). It is important to note that sarcopenia is associated with adverse health-related outcomes, including fall, fracture, and mortality (1, 2). Moreover, low handgrip strength is related to insulin resistance, hypertension, and mortality (26–28). We identified an inverse association between adherence to an anti-inflammatory nutrient pattern that was high in healthy oils, antioxidants, and anti-inflammatory nutrients and odds of sarcopenia and low handgrip strength. A cross-sectional study in Belgium revealed that sarcopenic subjects had a lower intake of lipid, iron, magnesium, and potassium (4). In the Maastricht Sarcopenia Study on adults aged 65 years or older, subjects with sarcopenia had 10–18% lower intake of n-3 fatty acids, vitamin B6, vitamin E, and magnesium compared with non-sarcopenic subjects (29). A cross-sectional study from UK Biobank cohort data, which was conducted on 68,002 adults age ≥60, indicated a significant positive link between dietary intake of oily fish and magnesium in either gender as well as dietary intake of iron and vitamin E in women and handgrip strength (30). In contrast to our findings, in Tasmanian older adults, dietary intakes of protein, iron, magnesium, and zinc were associated with muscle mass but not muscle strength (31). Overall, it seems that intake of anti-inflammatory nutrients may have a muscle protective role.

We found no association between the pro-Vit nutrient pattern and sarcopenia and its components. Although protein intake has been loaded in this pattern, we failed to find any significant association with sarcopenia. However, based on this finding, it is not true to claim that protein intake is not associated with sarcopenia because protein is only one component of this nutrient-based dietary pattern, and the combined effects of other nutrients loaded in this dietary pattern should be considered. For instance, lack of a significant association might be attributed to the loading of SFAs and cholesterol in this pattern. A longitudinal study in UK elderly people found that the “traditional British dietary pattern” characterized by a high intake of butter, red meat, gravy, and potato was associated with an increased risk of sarcopenia even when overall protein intake was good (32). However, a cross-sectional study in Belgium found that people with sarcopenia had significantly lower intakes of protein (4). As a conclusion, the meaning of a high-protein, nutrient-based dietary pattern can be different from the studies in which protein intake, as a sole nutrient, has been examined in relation to sarcopenia or its components.

In this study, adherence to the carbo-vit nutrient pattern that was characterized by high intakes of healthy carbohydrate, biotin, potassium, thiamin (B1), vitamin C, and chromium was associated with lower low gait speed. Low walking speed was related to cognitive impairment, dementia, cardiovascular diseases, mortality, disability, and hospitalization in earlier studies (33–36). In a cross-sectional analysis of community-dwelling Australian older adults aged ≥50 years with type 2 diabetes, greater adherence to the Mediterranean diet was significantly associated with better gait speed (37). In a cross-sectional study on 380 Spanish older adults aged 55–80, the investigators reported a positive association between the Mediterranean dietary pattern and walking speed and a negative link between the Western dietary pattern and walking speed (38). Therefore, dietary intake of some nutrients may have a beneficial role in muscle performance and gait speed.

Several mechanisms have been proposed for the role of nutrients in sarcopenia and its components. Oxidative stress has an important effect on the pathogenesis of sarcopenia (39). It causes atrophy, loss of myocytes, and muscle fibers by accumulation of mitochondrial and nuclear DNA damage (40, 41). Moreover, oxidative stress through increasing expression of inflammatory cytokines, such as interleukin- (IL-) 1, tumor necrosis factor (TNF), and IL-6 might damage muscle tissue (42, 43). Based on this background, antioxidant and anti-inflammatory nutrients, including vitamin E and C, MUFA, PUFA, omega-3, fiber, magnesium, caffeine, chromium, and copper, can play an important role against sarcopenia. Thiamine, pyridoxine, biotin, and iron are part of the coenzyme involved in energy production (44). Carbohydrates are the first source of energy for the muscles (44). Dietary intake of healthy carbohydrates high in fiber may have a beneficial effect on muscles. However, due to low data on nutrient intake and sarcopenia, additional investigation is needed in this area.

This study has several strengths. To our knowledge, this is the first study examining the association between nutrient-based dietary patterns and odds of sarcopenia and its components. Various confounding factors were adjusted for in the present analysis. Additionally, for the assessment of dietary intakes, a validated FFQ was used. Finally, valid definitions and valid tools were used for the diagnosis of sarcopenia. Along with strengths, some limitations should also be kept in mind. The cross-sectional design is the main limitation of our study, which prohibits us from conferring a causal relationship. The misclassification of study subjects and measurement error due to the use of FFQ is another limitation. It should also be kept in mind that muscle loss is different in men and women. One might be interested in a gender-stratified analysis to accurately examine the association of nutrient-based dietary patterns and low muscle mass. However, due to the small sample size and given that separating genders in the analysis resulted in a small number of people in each category, we did not perform such analysis. Last, because of financial limitations and inadequate access to the DEXA device in Tehran, the study was conducted on a small sample (maximum 300 cases). Thus, the generalization of these results to the whole Iranian population should be done with caution.

In conclusion, we observed that adherence to the anti-inflammatory nutrient pattern that is characterized by high consumption of polyunsaturated fat, monounsaturated fat, copper, vitamin E, omega-3, magnesium, iron, pyridoxine (B6), sodium, and caffeine might decrease the odds of sarcopenia and low muscle strength. Furthermore, the carbo-vit nutrient pattern with high amounts of fructose, glucose, dietary fiber, biotin, potassium, thiamin (B1), vitamin C, and chromium was associated with lower odds of low gait speed. Prospective studies are required to confirm these findings.

## Data Availability Statement

The raw data supporting the conclusions of this article will be made available by the authors, on reasonable request to the corresponding author.

## Ethics Statement

The studies involving human participants were reviewed and approved by Tehran University of Medical Sciences Ethics' Committee. The patients/participants provided their written informed consent to participate in this study.

## Author Contributions

AB, RHa, RHe, AM, and AE contributed to the conception, design, data collection, statistical analyses, data interpretation, manuscript drafting, approval of the final version of the manuscript, and agreed for all aspects of the work. All authors contributed to the article and approved the submitted version.

## Conflict of Interest

The authors declare that the research was conducted in the absence of any commercial or financial relationships that could be construed as a potential conflict of interest.
